# Heart Failure in Rheumatoid Arthritis: Epidemiology, Pathogenesis, Diagnosis, Treatment, and Emerging Insights—A Comprehensive Review

**DOI:** 10.3390/medicina62020380

**Published:** 2026-02-14

**Authors:** Goran Šukara, Josip Tečer, Ivana Jurin, Majda Golob, Marko Barešić, Joško Mitrović

**Affiliations:** 1 Division of Clinical Immunology, Allergology and Rheumatology, Department of Internal Medicine, Dubrava University Hospital, 10000 Zagreb, Croatia; gsukara@gmail.com (G.Š.); j.tecer@gmail.com (J.T.); mgolobmef@gmail.com (M.G.); 2 Department of Cardiovascular Diseases, Dubrava University Hospital, 10000 Zagreb, Croatia; ivanajurin1912@gmail.com; 3 Division of Clinical Immunology and Rheumatology, Department of Internal Medicine, Zagreb University Hospital Center, 10000 Zagreb, Croatia; marko.baresic@kbc-zagreb.hr; 4 School of Medicine, University of Zagreb, 10000 Zagreb, Croatia; 5 Faculty of Pharmacy and Biochemistry, University of Zagreb, 10000 Zagreb, Croatia

**Keywords:** arthritis, rheumatoid, heart failure, inflammation

## Abstract

Rheumatoid arthritis (RA) is a chronic, systemic inflammatory disease associated with an increased risk of cardiovascular complications, including heart failure (HF). HF represents a major cause of morbidity and mortality among patients with RA, contributing substantially to their reduced life expectancy. The early detection and optimal management of both traditional cardiovascular risk factors and RA-related inflammation are crucial to improving outcomes. In this comprehensive narrative review, we synthesize and critically appraise contemporary evidence on the epidemiology, pathophysiology, diagnosis, and management of HF in RA. We further explore emerging insights into the inflammatory and immune-mediated mechanisms driving myocardial dysfunction, advances in the early and preclinical detection of HF through novel imaging and biomarker approaches, and the evolving impact of modern RA therapies on cardiovascular health with a focus on heart failure. These developments highlight the importance of integrated, multidisciplinary strategies to prevent and manage heart failure in patients with rheumatoid arthritis.

## 1. Introduction

Rheumatoid arthritis (RA) is a chronic, systemic, progressive inflammatory disease characterized by symmetrical joint inflammation, progressive joint destruction, and diverse extra-articular manifestations. Patients with RA experience a substantially higher overall cardiovascular (CV) risk compared with the general population [1,2,3]. Among CV complications, heart failure (HF) is one of the most important causes of morbidity and mortality [4,5]. Importantly, this excess HF risk cannot be explained solely by traditional CV risk factors or by accelerated coronary artery disease. RA is associated with an increased risk of both HF with preserved ejection fraction (HFpEF) and HF with reduced ejection fraction (HFrEF). HFpEF usually occurs earlier in the disease course and in correlation with inflammatory activity [4,5,6,7,8,9].

Persistent systemic inflammation drives myocardial remodeling through fibrosis, hypertrophy, and increased myocardial stiffness, accompanied by endothelial and microvascular dysfunction [10,11]. These changes occur largely independently of atherosclerosis and contribute to diastolic dysfunction and subtle systolic dysfunction characteristic of HFpEF. Advanced echocardiographic techniques, cardiac magnetic resonance (CMR), and circulating cardiac biomarkers can detect these abnormalities even before clinical HF develops [11,12]. Chronic inflammation also accelerates atherosclerosis and leads to coronary artery disease, with the acute and/or chronic loss of cardiomyocytes and development of HFrEF [10,13]. Consequently, patients with RA tend to develop HF significantly earlier than individuals in the general population, with a markedly reduced life expectancy [14,15].

The early diagnosis of RA and effective treatment with the rational use of glucocorticoids and nonsteroidal anti-inflammatory drugs, optimized disease-modifying antirheumatic drug (DMARD) therapy, and aggressive management of traditional CV risk factors are essential to mitigate HF risk [16,17,18,19]. Likewise, the early recognition of subclinical myocardial dysfunction using biomarkers (e.g., natriuretic peptides) and modern imaging techniques may identify high-risk individuals who could benefit the most from targeted interventions [20,21].

The aim of this comprehensive narrative review is to provide an updated synthesis of emerging insights into the inflammatory and immune-mediated mechanisms driving myocardial dysfunction in RA, advances in the early and preclinical detection of HF through novel imaging and biomarker approaches, and the evolving influence of contemporary RA therapies on cardiovascular health, with a particular focus on heart failure.

## 2. Search Strategy, Study Selection and Review Design

We performed this review using the PubMed/MEDLINE and Google Scholar databases. A search was performed to identify the relevant literature published between January 2020 and September 2025. The search included combinations of keywords: “rheumatoid arthritis” AND “heart failure”; “rheumatoid arthritis” AND “heart failure” AND “epidemiology”; “rheumatoid arthritis” AND “heart failure” AND “pathogenesis”; “rheumatoid arthritis” AND “heart failure” AND “diagnosis”; and “rheumatoid arthritis” AND “heart failure” AND “treatment”. The titles and abstracts, and full texts, when necessary, of articles were screened, and relevant articles were selected for this comprehensive narrative review. The reference lists of relevant articles were also screened to identify additional studies. Only English-language, peer-reviewed, adult human studies were included. This review was designed as a comprehensive narrative review with the goal of providing clinically usable integration across epidemiologic, mechanistic, diagnostic, and therapeutic domains. When evidence was conflicting, particularly in the evaluation of therapeutic associations, we emphasized HF-specific endpoints, RA-relevant populations and dosing, and higher-quality population cohorts and meta-analyses while contextualizing discrepant findings according to study population, disease stage, drug dose and study design.

## 3. Review Findings

### 3.1. Epidemiology

The association between RA and HF was first demonstrated in population-based studies showing a nearly twofold higher HF incidence compared with the general population. Higher incidence was independent of conventional CV risk factors and coronary artery disease. In a study by Nicola et al. published in 2005, rheumatoid arthritis was detected as a significant risk factor for HF (hazard ratio (HR) 1.87, 95% confidence interval (CI) 1.47–2.39) after adjusting for demographics, ischemic heart disease, and CV risk factors [4]. The risk was higher among patients with RA who were rheumatoid factor (RF)-positive (HR 2.59, 95% CI 1.95–3.43) than among those who were RF-negative (HR 1.28, 95% CI 0.93–1.78). A study by Mantel et al. published in 2017 showed that in a cohort of patients with RA, the HRs were between 1.71 and 1.88 for the different HF subtypes [5]. The risk of non-ischemic HF increased rapidly after RA onset, in contrast to the risk of ischemic HF. High disease activity was associated with all HF types but was the most pronounced for non-ischemic HF.

Contemporary cohort studies have deepened our understanding of heart failure epidemiology in rheumatoid arthritis and highlighted evolving trends in its incidence over time. Myasoedova et al. reported, in a study published in 2023, in a population-based cohort spanning three decades, that the incidence of HF in patients with RA in the 2000s versus the 1980s was not statistically significantly different [22]. However, a closer examination of HF phenotypes demonstrated divergent trends: effect estimates for HFpEF in the 1990s and 2000s were about 20–30% higher and those for HFrEF were about 30% lower than the reference 1980s, signaling a possible evolving trend. This suggests a possible shift in the distribution of HF subtypes over time despite stable overall incidence. The reasons for this evolving trend are not fully understood. The improved detection and treatment of traditional cardiovascular risk factors and declining rates of ischemic heart disease likely contribute to the observed reduction in HFrEF. Conversely, increasing clinical awareness and diagnostic precision for HFpEF, together with the rising prevalence of HFpEF risk factors such as obesity, aging, and multimorbidity in the general population, may partially explain the relative increase in HFpEF. A similar pattern may be emerging within RA, in which chronic systemic inflammation and microvascular dysfunction predispose individuals to HFpEF.

A matched cohort study by Kawano et al. published in 2025 showed that HFpEF was the most common HF subtype in patients with RA and in controls (65% in RA vs. 59% in non-RA) [23]. It also showed that patients with RA had an HR of 1.79 (95% confidence interval [CI] 1.38–2.32) for incident HF when compared with those without RA and after adjusting for traditional CV risk factors. Again, patients with RA had a higher risk of HFpEF (HR 1.99, 95% CI 1.43–2.77), but there was no statistical difference in the HFrEF risk (HR 1.45, 95% CI 0.81–2.60). In 2025, Johnson et al. published the results of a large, retrospective, matched cohort study using data from 2000 to 2019 [24]. They matched 67,850 patients with RA (mean age 62.5, 87.1% male) to 570,933 non-RA controls (mean age 61.1, 85.8% male). RA was associated with an increased risk of HFpEF (adjusted hazard ratio (aHR) 1.51, 95% CI 1.46–1.57) and HFrEF (aHR 1.34, 1.30–1.38). HF risk was accentuated in RA patients with elevated inflammation and seropositive RA. No improvements in HF risk were observed over time (linear *p* for trend > 0.05 for all outcomes).

HFpEF appears to be the dominant phenotype in contemporary RA populations. Inflammatory activity and cumulative inflammatory disease burden correlate strongly with myocardial remodeling and dysfunction, even in the absence of overt heart disease. Ahlers et al., in a study published in 2020, demonstrated that elevated high-sensitivity C-reactive protein was independently associated with a greater risk of HF development in patients with RA, primarily of the HFpEF subtype, suggesting that chronic inflammation drives early myocardial remodeling [25]. A study by Alexandre et al. published in 2025 further showed that subclinical left ventricular systolic dysfunction was present in 24% of RA patients, with preserved left ventricular ejection fraction (LVEF) and no clinical signs or symptoms of HF, reinforcing that HFpEF predominates in early disease stages, while HFrEF usually emerges later [20].

Despite advances in cardiovascular care and rheumatoid arthritis therapy, the prognosis of HF in RA remains worse than in non-RA populations. In a study published in 2025, Chandrupatla and Singh reported that RA patients hospitalized for HF had significantly higher 90-day readmission rates (adjusted odds ratio (aOR) 1.16, 1.13–1.19) and greater in-hospital mortality (aOR 1.12, 1.04–1.20) compared with matched non-RA HF cohorts [26]. In the already mentioned study by Johnson et al., RA was associated with increased HFpEF-related mortality (aHR 2.05, 1.76–2.39), as well as HFrEF-related mortality (aHR 1.45, 1.29–1.63) [24].

Because RA predominantly affects women and HFpEF in the general population is also more common in women, sex may influence HF phenotype distribution in RA. However, available evidence indicates that increased HF risk in RA is not restricted to women. In the already mentioned large contemporary cohort in which over 80% of RA patients were men (veterans), RA remained associated with significantly higher risks of both HFpEF and HFrEF, suggesting that RA-related mechanisms driving HF extend across sexes [24].

Common comorbidities in RA, including obesity, diabetes, and chronic kidney disease, are established modifiers of HF risk (particularly HFpEF) and likely contribute to the observed subtype distributions. However, across major cohorts, the increased HF risk associated with RA generally persists after adjustment for traditional cardiovascular risk factors, indicating that comorbidities alone do not fully account for the excess risk [23,24,25].

In summary, as shown in Table 1, epidemiologic data from population registries and cohorts consistently show that RA confers a 1.5- to 2-fold increased risk of HF, with HFpEF predominating in early and inflammatory disease stages and HFrEF reflecting later ischemic damage. Still, there are no clear signs that improved disease control and advances in therapy have reduced the incidence of HF in patients with rheumatoid arthritis, especially of the HFpEF type. This likely reflects the combined impact of study cohorts whose follow-up spans earlier treatment eras before widespread treat-to-target care and before the broad availability and optimal use of biologic and targeted synthetic DMARDs, persistent cumulative inflammatory and glucocorticoid burden, and countervailing secular trends (aging, obesity, multimorbidity, and improved HFpEF recognition) that may offset gains from reduced ischemic disease. Also, mortality remains disproportionately high, further emphasizing the need for very early RA diagnosis, the aggressive suppression of inflammation, the aggressive treatment of traditional cardiovascular risk factors, and systematic cardiovascular surveillance.

### 3.2. Pathogenesis

Heart failure in RA is, at least in a significant part, an inflammatory, immune-mediated entity rather than merely a consequence of accelerated atherosclerosis [5,27]. Contemporary evidence further refines this concept, increasingly recognizing HF in RA as an inflammatory extra-articular manifestation with a predominantly HFpEF phenotype. Within this framework, two principal HF phenotypes can be distinguished in RA. The first is a non-ischemic, HFpEF-dominant phenotype, driven by chronic systemic inflammation, coronary microvascular dysfunction, and myocardial fibrosis. The second is an ischemic HFrEF phenotype, in which persistent inflammation accelerates atherosclerosis and promotes coronary artery disease, leading to acute or chronic cardiomyocyte loss and impaired systolic function. These two phenotypes often coexist and evolve along a continuum rather than as mutually exclusive entities.

Proinflammatory cytokines characteristic of RA, such as tumor necrosis factor (TNF), interleukin-1 (IL-1) and interleukin-6 (IL-6), have been implicated in impaired cardiomyocyte contractility, fibroblast activation, and apoptosis in experimental and translational studies, changes that contribute to myocardial hypertrophy, stiffness, and diastolic dysfunction [13,27]. Concomitant endothelial activation and microvascular dysfunction mediated by reduced nitric oxide bioavailability, oxidative stress, and leukocyte–endothelium interactions limit myocardial perfusion and promote ischemia and fibrosis even in the absence of obstructive coronary disease [13]. Cardiac MR (magnetic resonance) studies provide in vivo confirmation of diffuse myocardial edema and fibrosis in RA patients with preserved ejection fraction (EF), establishing the myocardium as a direct inflammatory target [12].

Recent data have not overturned this model but have refined and strengthened it at several levels. On the mechanistic side, Chen et al. provided an updated synthesis of how inflammatory pathways in RA translate into cardiac dysfunction, emphasizing not only classic proinflammatory cytokines but also the failures of pro-resolving lipid mediators, persistent macrophage activation, and neutrophil extracellular traps as drivers of cardiomyocyte injury, endothelial dysfunction, and fibrosis [10]. Park and Bathon integrated these and other data into a comprehensive framework that links RA-specific pathways, synovial cytokine networks, and microvascular inflammation to the clinical spectrum of myocardial dysfunction and HF subtypes in RA [11].

Additional immune pathways (e.g., Th17 pathway, inflammasome activation) and emerging biomarkers of fibrosis/endothelial dysfunction have been proposed as contributors to RA-related myocardial injury but remain incompletely defined for clinical application [10,11].

An RA-HF study by Ferreira et al. added a molecular layer to the link between RA and HF by demonstrating that HF in unselected RA patients is accompanied by a distinct proteomic signature enriched for inflammatory, neurohormonal and matrix-remodeling proteins, including elevated adrenomedullin and TNF receptor superfamily members, thereby further linking systemic immune activation to myocardial dysfunction in RA [28]. In parallel, Norouzi et al.’s work on anti-MCV (modified citrullinated vimentin) and the broader literature on ACPA (anti-citrullinated protein antibodies)-related myocardial effects support the idea that specific autoantibody profiles may identify individuals with heightened susceptibility to immune-mediated myocardial injury [29].

Finally, genetic studies using Mendelian randomization provide evidence supporting a possible causal contribution of rheumatoid arthritis to heart failure. Two independent analyses have reported that genetically proxied susceptibility to RA is associated with an increased risk of HF in European ancestry cohorts [30,31].

The HFrEF phenotype in RA reflects the synergistic interaction between chronic inflammation and accelerated atherosclerosis. RA promotes plaque formation, destabilization, and thrombosis through endothelial dysfunction, altered lipid metabolism, and sustained inflammatory activation. The resulting coronary artery disease leads to myocardial infarction (MI), cardiomyocyte loss, and progressive systolic dysfunction. However, even in this ischemic pathway, inflammation remains a central amplifier of myocardial damage [1,4,5,13].

**Figure 1:**
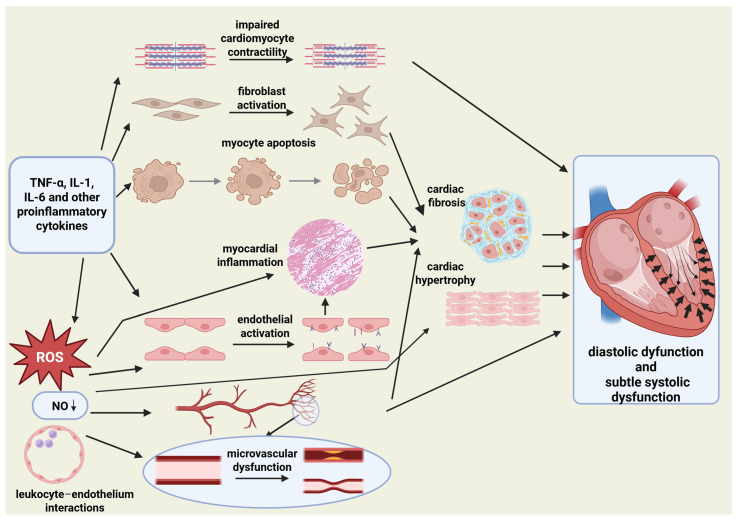
Inflammation-driven pathogenesis of HFpEF in rheumatoid arthritis. Schematic diagram illustrating proposed mechanisms linking systemic inflammation in rheumatoid arthritis to myocardial remodeling and diastolic dysfunction with subtle systolic dysfunction characteristic of HFpEF. Abbreviations: TNF-α, tumor necrosis factor alpha; IL, interleukin; ROS, reactive oxygen species; NO, nitric oxide. Created in BioRender. Tečer, J. (2026) https://BioRender.com/9igkn8p accessed on 29 January 2026).

Collectively, these data establish HF in RA as a complex inflammatory cardiomyopathy in which immune-mediated myocardial injury, coronary microvascular dysfunction, interstitial fibrosis, and adverse ventricular remodeling converge to produce a predominantly HFpEF phenotype (Figure 1), with HFrEF emerging as a later ischemic complication. This paradigm has profound implications for early diagnosis, risk stratification, and therapeutic targeting in RA-associated heart failure.

### 3.3. Diagnosis: Subclinical Disease, Imaging, and Biomarkers

Cardiac involvement in RA precedes clinical HF, often progressing silently through diffuse myocardial inflammation and interstitial fibrosis with progressive diastolic dysfunction and subtle systolic impairment. Traditional cardiovascular risk assessment is insufficient to detect these early changes, and contemporary evidence increasingly supports a targeted, multimodal diagnostic strategy focused on identifying subclinical myocardial disease, particularly the HFpEF-like phenotype that dominates in this population. Recent studies using advanced echocardiography, cardiac magnetic resonance (CMR), and biomarkers have significantly refined the ability to detect myocardial involvement long before symptoms occur.

#### 3.3.1. Imaging

##### Echocardiography

Transthoracic echocardiography remains the cornerstone of heart failure diagnosis and functional cardiac assessment. In RA, advanced echocardiography allows for the detection of subtle structural and functional myocardial abnormalities, long before the development of overt HF. In a cohort study of RA patients without known cardiovascular disease, Rodrigues et al. demonstrated that 17% of patients exhibited subclinical left ventricular (LV) systolic or diastolic dysfunction, despite being asymptomatic [32]. Abnormal ventricular function correlated with higher N-terminal pro-B-type natriuretic peptide (NT-proBNP) levels and impaired exercise capacity, underscoring that clinically silent myocardial disease in RA is neither rare nor benign. In a longitudinal imaging study by Park et al., diastolic dysfunction was found in 40.7% of RA patients at baseline, rising to 57.9% after 4 to 6 years of follow-up [33]. These changes correlated with baseline RA disease activity and cardiac biomarkers (brain natriuretic peptide (BNP), troponin-I), highlighting the inflammation-driven progression of myocardial impairment and diastolic dysfunction.

Speckle-tracking echocardiography with the measurement of global longitudinal strain (GLS) allows for the early detection of subclinical systolic dysfunction. In a prospective cohort study published by Alexandre et al. in 2025, it was demonstrated that GLS detects subclinical LV systolic dysfunction in asymptomatic patients even when ejection fraction is normal [20]. Importantly, impaired GLS was a strong independent predictor of major adverse cardiovascular events, including HF hospitalization and CV death, outperforming conventional echocardiographic parameters such as left ventricular ejection fraction. These findings place GLS at the center of early diagnostic algorithms for RA-related myocardial disease. Taken together, current evidence supports the early use of echocardiography in patients with RA, where comprehensive diastolic evaluation can detect early HFpEF pattern pathology, and also GLS for the sensitive detection of early systolic dysfunction. These methods enable the very early risk stratification of patients with RA for the development of clinically overt HF.

##### Cardiac Magnetic Resonance

Cardiac magnetic resonance provides detailed tissue characterization and has been transformative in defining the myocardial changes in RA. In a study published in 2015, Ntusi et al. demonstrated MR findings of diffuse myocardial inflammation and diffuse or focal fibrosis, findings that were present despite preserved LVEF in patients with no known heart disease [12]. These observations have been expanded significantly by contemporary studies. In a study published in 2021, Malczuk et al. evaluated young (median age 41 years; 83% female) RA patients without known cardiovascular disease [34]. Using cardiac magnetic resonance, they identified features of myocardial edema without signs of myocardial fibrosis in 39% of young RA patients. The presence of myocardial edema was associated with radiographic joint erosions and a longer delay between symptom onset and diagnosis, which suggests that early diagnosis and effective treatment initiation may prevent not only joint destruction but also myocardial damage. These results underscore that inflammatory myocardial injury begins early and may be independent of traditional risk factors.

Comprehensive and detailed insights come from a 2025 analysis by Tarjanyi et al. [21]. They demonstrated significant myocardial alterations in RA patients, including significantly increased left ventricular mass, impaired systolic function, and adverse ventricular remodeling, when compared with healthy controls, independent of confounding factors such as age, sex, and conventional CV risk factors. They also revealed a significant correlation between the Disease Activity Score in 28 joints (DAS28) score and the decline in the left ventricular global function index (LVGFI) in patients with RA.

A study by Koivuniemi et al. published in 2021 showed that myocardial abnormalities could be modifiable with effective RA therapy [35]. In a prospective CMR follow-up, it was shown that compared with controls, 58 RA patients had a slightly lower ventricular function, although in the normal range, and a longer T1 time at baseline. None of the control patients with fibromyalgia had late gadolinium enhancement (LGE), but this was frequent in RA (67%). After one year of DMARD treatment, DAS28-CRP declined, and ventricular function tended to improve, but the number of patients with LGE remained unchanged. However, the number of LGE-positive heart segments either decreased or stayed the same in 91% of RA patients. They concluded that in early RA patients, achieving tight remission was associated with LGE stabilization, after adjustment for age, metabolic syndrome, baseline inflammatory activity, and level of physical activity. This provides compelling evidence that RA-associated myocardial disease is driven by active inflammation and may respond to early and aggressive disease control.

Cardiac computed tomography, particularly coronary calcium scoring or CT angiography, may also be considered for the assessment of atherosclerotic burden in selected RA patients at high cardiovascular risk, although its role relates primarily to ischemic risk stratification rather than the detection of inflammatory myocardial dysfunction or HF phenotype characterization.

#### 3.3.2. Biomarkers

Circulating cardiac biomarkers play a central role in heart failure diagnosis in the general population, but their utility in detecting subclinical myocardial disease in RA is more nuanced. In the RA-HF study published in 2021, Ferreira et al. showed that the level of NT-proBNP was significantly higher in patients with established HF, within the RA population [28]. NT-proBNP is therefore valuable for identifying RA patients with significant hemodynamic stress or established HF. However, contemporary studies show that natriuretic peptides are not sufficiently sensitive to detect subclinical myocardial involvement. In the Porto-RA cohort study published by Alexandre et al., NT-proBNP was markedly less effective than GLS in identifying early LV dysfunction [20]. Similarly, in the diastolic dysfunction study by Park et al., BNP and troponin-I were associated with individual echocardiography-based parameters of diastolic function (left atrial volume index and E/e′) but not with baseline composite diastolic dysfunction [33]. These findings indicate that natriuretic peptides should be viewed as adjunctive rather than primary screening tools in asymptomatic RA patients.

Several additional inflammatory and other biomarkers (e.g., IL-6, FGF-23, and markers of fibrosis or endothelial dysfunction) are being explored in research settings, but their role in clinical HF risk stratification in RA remains unvalidated.

In summary, the diagnosis of HF in RA is undergoing a paradigm shift, from the late recognition of overt HF to the early detection of subclinical, inflammatory myocardial disease with the use of advanced imaging methods and biomarkers. This refined diagnostic approach offers the potential to intervene earlier, modify disease trajectory, and ultimately prevent progression to clinically overt HF.

The practical implementation of advanced cardiac diagnostics in RA remains challenging. Techniques such as speckle-tracking echocardiography and CMR require specialized equipment, software, and operator expertise, which may not be uniformly available outside tertiary centers. CMR, in particular, is resource-intensive, with limitations related to cost, scanner availability and examination time. Biomarkers such as natriuretic peptides are widely accessible but lack specificity for early inflammatory myocardial disease, limiting their role as standalone screening tools. These constraints underscore the need for pragmatic risk-stratified approaches and future studies defining which RA subgroups derive the greatest clinical benefit from advanced cardiac evaluation.

### 3.4. Treatment and Prevention: Effects of RA Therapies on Heart Failure Risk

Therapeutic strategies in rheumatoid arthritis exert a major influence on cardiovascular outcomes. Across drug classes, a consistent principle has emerged that the effective, sustained suppression of systemic inflammation reduces cardiovascular complications, whereas chronic glucocorticoid exposure increases them [36,37,38,39]. Heart failure-specific data remain limited for many DMARD classes; however, studies and analyses from 2020 to 2025 have substantially refined our understanding of how contemporary RA therapies shape HF risk. For several therapies, HF-specific outcomes are reported less frequently than composite cardiovascular endpoints; therefore, HF-related interpretations are based on the most specific available data and, where necessary, contextualized within broader cardiovascular findings. Because much of the HF-related therapeutic literature is observational and subject to confounding by indication, we interpret associations cautiously and emphasize consistency across study designs and endpoints rather than causal inference.

#### 3.4.1. Glucocorticoids

Glucocorticoids still have an important role in the rapid control of inflammation in early or flaring RA, yet they pose well-established cardiovascular risks. Ocon et al., in a study published in 2021, demonstrated a clear, dose-dependent cardiovascular hazard associated with glucocorticoid therapy in RA [17]. In glucocorticoid-naive patients, starting prednisone at ≥5 mg/day significantly increased short-term major cardiovascular events, including heart failure, while ≤4 mg/day showed no detectable excess risk. These associations persisted after adjusting for disease activity, confirming that even brief exposure to moderate-dose glucocorticoids confers early cardiovascular vulnerability. In a population-based cohort study of 87,794 patients with immune-mediated inflammatory diseases (including 25,324 with RA), Pujades-Rodriguez et al. showed a clear, dose-dependent increase in cardiovascular risk with oral glucocorticoids [40]. Even <5 mg prednisolone equivalent daily almost doubled overall cardiovascular disease (CVD) risk (HR 1.74, 95% CI 1.64–1.84), with 1-year CVD incidence rising from 1.5% (no steroids) to 3.8% (<5 mg) and 9% (≥25 mg). Across immune-mediated diseases, heart failure and acute myocardial infarction showed the highest glucocorticoid dose effect estimates; disease-specific analyses, including those of RA, demonstrated similarly strong dose-dependent increases in risk.

Although glucocorticoids suppress inflammatory activity, several mechanisms plausibly explain their association with increased HF risk. Glucocorticoids can promote sodium and water retention and increase intravascular volume, thereby raising cardiac preload and precipitating congestion in susceptible individuals. They also contribute to hypertension via mineralocorticoid receptor activity and vascular effects and worsen cardiometabolic risk by inducing insulin resistance, weight gain, and dyslipidemia, which can accelerate myocardial stress and adverse remodeling. These hemodynamic and metabolic effects provide a biologically plausible explanation for the consistent dose-dependent increases in heart failure and broader cardiovascular risk observed in contemporary cohorts of RA and other immune-mediated inflammatory diseases [17,40].

#### 3.4.2. Conventional Synthetic DMARDs (csDMARDs)

##### Methotrexate

Methotrexate (MTX) remains the anchor therapy in RA and, according to available data, possesses the strongest cardiovascular safety profile among csDMARDs. Across contemporary cohorts and meta-analyses, methotrexate shows the strongest evidence for HF protection among csDMARDs. In a large retrospective cohort study, Ahlers et al. reported a 21% higher HF risk in rheumatoid arthritis patients compared with controls, but MTX use within the RA group was associated with an approximately 25% lower incident HF risk, independent of traditional risk factors and inflammatory markers [25]. In a multicenter, prospective cohort study by Johnson et al., MTX reduced composite cardiovascular events by 24% and HF hospitalization by 57% (HR 0.43, 95% CI 0.24–0.77), and mediation analyses showed that this benefit was only partially explained by improvements in RA disease activity [16]. These findings suggest alternative MTX-related mechanisms that may modify CVD risk in rheumatoid arthritis. A meta-analysis by Sun et al. published in 2021 including 10 studies and 195,416 RA patients demonstrated that MTX reduced composite cardiovascular events (relative risk (RR) 0.80, 95% CI 0.73–0.88), with HF included among the prevented outcomes [41]. In a large multi-institutional network analysis by Cervantes et al., MTX users experienced fewer CV events, particularly congestive HF, compared with MTX-naive RA patients ([42]—abstract). Untreated RA patients were 11% more likely to develop myocardial infarction (RR 1.11, 95% CI (1.09, 1.14), *p*-value < 0.0001) and 14% more likely to develop chronic ischemic heart disease (RR 1.14, 95% CI (1.13, 1.15), *p*-value < 0.0001). HF incidence was 21% higher (RR 1.21, 95% CI (1.19, 1.22), *p*-value < 0.0001) in the untreated group. As most MTX-related cardiovascular and HF data are observational, these associations may be influenced by confounding by indication and differences in baseline disease severity and should therefore be interpreted as associative rather than definitively causal. Collectively, these findings support MTX as the preferred anchor therapy for RA patients with elevated HF risk, where not contraindicated.

##### Hydroxychloroquine

The cardiovascular profile of hydroxychloroquine (HCQ) has been somewhat clarified in several recent studies. Sorour et al., in a nested case–control study published in 2021, found no association between HCQ exposure and incident HF in RA overall [43]. HCQ cumulative dose was not associated with HF (odds ratio (OR) 0.96 per 100 g increase in cumulative dose, 95% CI 0.90–1.03). Also, no association was found for patients with a cumulative dose ≥ 300 g (OR 0.92, 95% CI 0.41–2.08). The duration of HCQ intake prior to HF was also not associated with HF (OR 0.98, 95% CI 0.91–1.05). In 2022, D’Andrea et al. published a large comparative analysis of patients with RA older than 65 years starting methotrexate or hydroxychloroquine that reported that hydroxychloroquine was not associated with the risk of sudden cardiac arrest or ventricular arrhythmia (SCA/VA) (HR: 1.03; 95% CI: 0.79–1.35) or major adverse cardiovascular events (MACE) (HR: 1.07; 95% CI: 0.97–1.18) compared with methotrexate [44]. Conversely, in patients with a history of HF, hydroxychloroquine initiators showed a higher risk of MACE (HR: 1.30; 95% CI: 1.08–1.56), cardiovascular mortality (HR: 1.34; 95% CI: 1.06–1.70), all-cause mortality (HR: 1.22; 95% CI: 1.04–1.43), myocardial infarction (HR: 1.74; 95% CI: 1.25–2.42), and hospitalized HF (HR: 1.29; 95% CI: 1.07–1.54) compared with methotrexate initiators. Cardiovascular risks were not different in patients without a history of HF except for an increased hospitalized HF risk (HR: 1.57; 95% CI: 1.30–1.90) among hydroxychloroquine initiators. Conversely, Iyer et al., in a study published in 2023, found that HCQ use was associated with lower all-cause mortality in older RA patients, though without reductions in major CV events [45]. Taken together, at this point, HCQ appears neutral from an HF standpoint and only possibly harmful in older patients with a history of HF. Based on all available evidence, it should not be used for cardiovascular protection in patients with rheumatoid arthritis.

For sulfasalazine and leflunomide, HF-specific data remain insufficient.

HF-specific data are also insufficient for csDMARD combination regimens (e.g., methotrexate plus leflunomide), as HF outcomes are infrequently reported; therefore, no HF risk inference can be made for combination therapy.

#### 3.4.3. Biologic DMARDs

##### TNF Inhibitors

Prospective HF trials outside RA demonstrated that high-dose TNF blockade can worsen outcomes in established HF. In ATTACH, patients with NYHA (New York Heart Association) III-IV HF receiving infliximab 10 mg/kg had an increased combined risk of death or HF hospitalization (HR 2.84, 95% CI 1.01–7.97) compared with placebo, without clinical benefit [46]. Similarly, the combined RECOVER/RENAISSANCE program (RENEWAL), including ≈ 2000 patients with NYHA II-IV HF and reduced ejection fraction, showed no improvement with etanercept and a non-significant trend toward harm (RR 1.10, 95% CI 0.91–1.33) [47]. These pivotal findings underpin current caution against tumor necrosis factor inhibitors (TNFis) in advanced HF.

In contrast, RA-focused studies using standard TNFi dosing show no comparable harm. A nationwide Taiwanese cohort of csDMARD refractory RA (*n* = 3210) reported that the incidence rates of hospitalization for HF for the TNF-α inhibitor and csDMARD groups were 3.66 and 4.72 per 1000 person-years, respectively (adjusted hazard ratio (aHR) 0.59; 95% confidence interval (CI) 0.35–0.97), and the results remained consistent in patients with a history of HF (aHR 0.66; 95% CI 0.03–14.46) and without (aHR 0.49; 95% CI 0.27–0.89) [48]. The findings suggest that those who switched to TNF-α inhibitors had a reduced risk of HF compared with those who switched to another csDMARD regimen. In a large-scale meta-analysis published in 2025 by Galajda et al., 45 studies were included [49]. They compared patient groups with immune-mediated inflammatory diseases receiving TNFis to non-TNFi-exposed controls. The outcome was the incidence of worsening, de novo, and composite HF. For the worsening of HF, the pooled results of non-randomized studies showed no statistically significant risk-increasing effect of TNFis (RR 1.18, 95% CI: 0.69–2.00). Analyses from both RCTs and non-randomized data indicated no increased risk of de novo HF in the TNFi group compared with controls (RR 0.87, 95% CI: 0.60–1.25 and RR 0.86, 95% CI: 0.64–1.14, respectively). Similarly, no increased risk was found for composite (worsening and de novo) HF in the TNFi-treated group versus controls, pooling non-randomized data.

Taken together with extensive pre-2020 RA data [50,51,52], contemporary evidence suggests that the adverse signals seen in non-RA HF trials likely reflect dosing and disease stage effects rather than class toxicity. Current evidence suggests that in RA, TNFis do not increase HF incidence and may confer protection when used to achieve adequate inflammatory control.

Still, and consistent with all evidence, RA treatment guidelines recommend using a non-TNF inhibitor biologic rather than a TNFi in patients with NYHA III–IV HF and to switch from the TNFi if HF develops while allowing for cautious use in patients with mild or pre-HF when they represent the most effective option for disease control.

##### IL-6 Inhibitors

Data for IL-6 inhibitors are relatively scarce and remain limited. In a large US multi-database cohort of RA patients who had previously received another biologic drug or tofacitinib, the initiation of tocilizumab was not associated with an increased risk of major adverse cardiovascular events (hospitalized myocardial infarction or stroke) compared with TNF inhibitors; heart failure events were not specifically analyzed [53]. A systematic review and network meta-analysis concluded that tocilizumab has cardiovascular outcomes (MACE, MI, stroke) comparable to other bDMARDs (biologic DMARDs), although there were not enough data available to perform a network meta-analysis on congestive heart failure as an outcome [54]. Long-term extension studies, totaling 12,293 patient-years of tocilizumab exposure, revealed no new safety signals, with cardiovascular events remaining uncommon [55]. In a national cohort of TNFi non-responders, switching to second-line tocilizumab or abatacept was associated with lower rates of major cardiovascular events compared with rituximab [18]. For sarilumab, data are even more limited, with available pooled safety analyses that describe the low and stable incidence of major adverse cardiovascular events and no emerging heart failure safety signal, albeit with shorter follow-up and limited HF-specific data [56]. Overall, IL-6 inhibitors do not appear to confer excess major cardiovascular risk and are generally considered acceptable in RA patients with coexisting heart failure, particularly when TNF inhibitors are avoided, while recognizing the limited HF-specific evidence.

##### Rituximab

Evidence on heart failure with rituximab in rheumatoid arthritis remains limited. In the global rituximab clinical trial program and its 9.5-year extension (>11,000 patient-years), serious cardiovascular events were uncommon, myocardial infarction and stroke rates remained comparable with background RA risk, and no cumulative signal for cardiac failure emerged, although HF was not systematically predefined as an endpoint [57]. The SUNSTONE observational registry similarly demonstrated low and stable rates of cardiovascular/thrombotic events over a mean 3.9-year follow-up, without increasing risk over repeated exposure cycles [58]. By contrast, a Taiwanese national cohort of TNF inhibitor non-responders found higher major cardiovascular event rates with rituximab, and abatacept was associated with significantly lower heart failure risk (HR 0.20; 95% CI 0.05–0.83) [18]. Expert reviews have not identified convincing evidence of rituximab worsening cardiac function in RA; however, rare case reports and precautionary guidance suggest cautious use in patients with pre-existing or unstable heart failure [59,60].

##### Anakinra

Heart failure data for anakinra in rheumatoid arthritis are very limited. Randomized and long-term extension trials in high-risk RA cohorts report very low cardiac event rates and no signal for incident or worsening HF [61,62]. A systematic review of >2800 patients confirmed no excess cardiovascular harm, although HF was not a predefined endpoint [63]. Overall, based on available limited data, anakinra appears cardiovascularly neutral, with no evidence to avoid its use solely due to HF.

##### Abatacept

Abatacept consistently demonstrates one of the most favorable cardiovascular safety profiles among RA biologics. Clinical trial and consensus reports cite no evidence of worsening HF, with caution only in advanced (NYHA IV) HF [64,65]. A large real-world US cohort showed a lower risk of cardiovascular events with abatacept versus TNF inhibitors in patients with or without pre-existing CVD [66]. In TNF inhibitor non-responders, abatacept reduced major cardiovascular events, including HF, compared with rituximab [18]. Thus, abatacept is often regarded as a preferred biologic option when HF coexists with RA.

#### 3.4.4. JAK Inhibitors

Heart failure-specific evidence for JAK inhibitors in RA is relatively sparse. Pooled baricitinib analyses demonstrate stable rates of major cardiovascular events and no progressive HF signal [67]. Integrated upadacitinib safety data show a MACE incidence comparable to adalimumab or methotrexate, without the suggestion of HF risk [68]. Conversely, the ORAL Surveillance Trial revealed higher major cardiovascular event rates with tofacitinib versus TNF inhibitors among older RA patients with CV risk factors, prompting class-wide warnings, although HF was not prespecified as an endpoint [69]. Post-ORAL Surveillance data have shifted even greater attention toward cardiovascular safety across the JAK inhibitor class. Real-world STAR-RA study data showed no overall excess CV risk with tofacitinib but suggested caution in patients with established cardiovascular disease [70]. Subsequent real-world analyses have reported heterogeneous cardiovascular risk signals across populations and comparators; however, these studies primarily evaluate composite cardiovascular endpoints rather than heart failure specifically [71,72,73]. Importantly, HF outcomes remain infrequently reported and rarely adjudicated in both clinical trials and observational cohorts, and current evidence does not permit the reliable differentiation of HF risk among individual JAK inhibitors. Thus, JAK inhibitors should be used carefully in RA patients with HF or high CV risk, with a preference for alternative biologic when feasible.

Based on current evidence, also shown in Table 2, the HF-conscious management of RA should aim to minimize glucocorticoids and avoid chronic doses ≥ 5 mg/day, prefer MTX-based therapy as the first-line treatment for its consistent cardioprotective profile, use HCQ cautiously in patients with existing HF or high HF risk, and escalate promptly to effective biologic therapy (TNFi or non-TNFi) when MTX fails, prioritizing tight inflammatory control. In established HFrEF, abatacept, rituximab, or IL-6 inhibition should be considered preferred biologic options. For JAK inhibitors, use in patients with high CV risk should be restricted unless no suitable alternatives exist.

Overall, the weight of evidence supports an approach in which the aggressive, steroid-sparing control of RA inflammation is central to preventing HF, while therapy selection is tailored to individual cardiovascular risk profiles.

## 4. Discussion

This comprehensive narrative review synthesizes emerging evidence on the complex relationship between rheumatoid arthritis and heart failure, highlighting that HF in RA is not simply an epiphenomenon of accelerated atherosclerosis but a predominantly inflammatory, immune-mediated manifestation with a strong HFpEF signature [4,5,7,11,23,24,25]. Across epidemiologic, diagnostic, and therapeutic domains, recent data converge on a consistent message that despite advances in RA management and cardiovascular care, the risk and prognosis of HF in RA remain unacceptably poor, particularly for HFpEF, and are tightly linked to cumulative inflammatory burden rather than to traditional risk factors alone [4,5,6,7,22,23,24,25,26].

Several aspects distinguish this review from prior work on cardiovascular disease in RA. First, it concentrates specifically on HF, rather than broader composite CV endpoints, and integrates the most recent data up to 2025. Second, it brings together mechanistic, imaging, biomarker, and genetic evidence to frame HF in RA as a primarily inflammatory HFpEF-like entity, supported by CMR evidence of diffuse myocardial edema and fibrosis, proteomic signatures, autoantibody associations, and Mendelian randomization data [12,21,28,29,30,31]. Third, this review emphasizes subclinical myocardial disease and offers a practical, though still conceptual, framework for early detection that combines comprehensive echocardiography (including GLS), CMR, and the selective use of biomarkers, shifting the focus from late-stage HF to preclinical cardiomyopathy [20,21,32,33,34,35]. Finally, it systematically appraises HF-specific and broader CV data across RA therapies, translating them into a steroid-sparing, HF-conscious treatment strategy that can be operationalized in daily practice [16,17,18,36,37,38,39,40,41,42,43,44,45].

### 4.1. Clinical Perspective

From a clinical perspective, these insights argue strongly for a more proactive, integrated cardiorheumatology approach. Rheumatologists and cardiologists should jointly identify RA patients at the highest risk and consider targeted cardiac screening with echocardiography and MR where appropriate [11,20,21,27]. The concept of early heart failure-directed pharmacotherapy, including RAAS (renin–angiotensin–aldosterone system) blockade or SGLT2 (sodium–glucose cotransporter 2) inhibition, in RA patients with subclinical myocardial abnormalities is biologically plausible and supported by extrapolation from non-RA HF populations, although direct evidence in RA remains limited [74,75,76,77,78,79]. This therefore represents a critical priority for future prospective interventional trials. These developments support the creation of joint rheumatology–cardiology consensus pathways to guide risk stratification, screening, and HF management in RA, with the long-term aim of developing evidence-based clinical algorithms as data mature.

### 4.2. Research Perspective

From a research perspective, several major knowledge gaps remain. These include the standardized definitions and diagnostic algorithms for RA-related HFpEF and preclinical myocardial disease, prospective studies testing whether systematic cardiac screening improves outcomes, head-to-head comparative effectiveness studies of biologic and targeted synthetic DMARDs with HF-specific endpoints, and mechanistic studies linking specific inflammatory and autoantibody profiles to myocardial phenotypes, potentially enabling personalized risk stratification. Future studies should also investigate whether RA-specific genetic susceptibility markers, such as the HLA-DRB1 shared epitope, contribute to differential susceptibility to heart failure and myocardial phenotypes in RA. In parallel, prospective trials are needed to determine whether the early initiation of HF-directed therapies, including SGLT2 inhibitors or mineralocorticoid receptor antagonists, in RA patients with subclinical myocardial dysfunction improves cardiovascular outcomes.

### 4.3. Limitations

The conclusions of this review must be interpreted in light of several important limitations. Most of the available epidemiologic and therapeutic data are observational, susceptible to residual confounding, confounding by indication, and the misclassification of HF phenotypes. The epidemiologic literature synthesized is predominantly derived from North American and European cohorts and may therefore not fully represent global populations. HF definitions and adjudication methods vary across cohorts. HFpEF was frequently operationalized using EF thresholds rather than uniform contemporary HFpEF criteria incorporating diastolic indices and biomarkers, and temporal increases in HFpEF may partly reflect evolving ascertainment and diagnostic practice. Imaging studies are frequently single-center, with relatively small sample sizes and potential selection bias toward patients with higher disease activity or suspected CV disease. Also, this is a comprehensive rather than systematic synthesis, limited to English-language, adult human studies and focused on publications from 2020 to 2025 supplemented by key earlier foundational work. As such, some relevant studies may not have been captured, and the relative weight assigned to individual findings reflects expert judgment rather than formal meta-analysis. A systematic approach was not pursued because key review objectives required the cross-domain integration of heterogeneous study designs (mechanistic, imaging, biomarker, and therapeutic data) where standardized outcome harmonization and formal pooling were not consistently feasible.

## 5. Conclusions

Heart failure in rheumatoid arthritis represents a major and under-recognized determinant of morbidity and mortality, increasingly characterized by an inflammatory HFpEF phenotype that arises from chronic systemic and microvascular inflammation, autoimmune myocardial injury, and maladaptive remodeling [4,5,11,12,13,21,23,25,27]. Contemporary evidence indicates that HF risk in RA has not significantly improved despite advances in disease-modifying therapy and that traditional CV risk management alone is insufficient [13,22,23,24].

This comprehensive narrative review highlights that the early, aggressive, and steroid-sparing control of RA inflammation should be central to HF prevention [16,17,18,36,37,38,39,40,41]. At the same time, there is a pressing need to shift from the late recognition of overt HF to the intentional detection of subclinical myocardial disease using advanced echocardiography, CMR, and selected biomarkers, particularly in high-risk patients [12,20,21,32,33,34,35].

Embedding these insights into routine practice will require closer collaboration between rheumatologists and cardiologists, the development of pragmatic screening algorithms, and interventional studies testing whether the early identification and treatment of RA-related myocardial disease can alter the trajectory of HF. Until such data are available, clinicians should regard HF prevention and surveillance as integral components of comprehensive RA care, rather than secondary considerations.

## Figures and Tables

**Table 1:** Epidemiology of heart failure in rheumatoid arthritis: major contemporary studies. Summary of key population-based investigations evaluating heart failure incidence, subtype distribution, and outcomes in rheumatoid arthritis.

Study/Year	Design and Population	Sample Size/Follow-Up	Key Findings	Implications
Nicola et al., 2005 [4]	Population-based retrospective cohort	575 RA patients, 583 controls; 46-year span	HF risk nearly doubled vs. non-RA (HR 1.87). RF-positive RA had greatest risk (HR 2.59).	RA confers substantial HF risk; seropositivity amplifies risk.
Mantel et al., 2017 [5]	Nationwide cohort study	>46,000 RA cases; 500,000 controls; median follow-up ~5 years	RA increased risk of ischemic and non-ischemic HF (HR 1.71–1.88). Risk rose rapidly after RA onset; strongest association for non-ischemic HF.	HF risk in RA is not solely atherosclerotic; inflammatory HF phenotype likely.
Myasoedova et al., 2023 [22]	30-year population-based cohort	905 RA cases; population-based cohort across three decades	Overall HF incidence unchanged across eras; HFpEF effect estimates ↑ 20–30%, HFrEF ↓ 30% vs. 1980s.	HF phenotype distribution may be shifting despite stable incidence.
Kawano et al., 2025 [23]	Retrospective matched cohort	4873 RA vs. 48,730 controls; median follow-up 7.4 years	Higher HF incidence in RA (HR 1.79). HFpEF strongest signal (HR 1.99); HFrEF non-significant (HR 1.45).	HFpEF predominates in RA; non-ischemic cardiac remodeling appears dominant pathway.
Johnson et al., 2025 [24]	Large administrative database/matched cohort	67,850 RA vs. 570,933 controls; 2000–2019	RA associated with increased risk of HFpEF (aHR 1.51) and HFrEF (aHR 1.34). No temporal decline in HF risk.	Persistent HF burden in RA despite modern therapy; no evidence of improving trends.
Ahlers et al., 2020 [25]	Prospective cohort evaluating inflammation	9889 RA patients vs. 9889 controls; ~177,566 person-years of follow-up	Elevated CRP independently predicted HF, particularly HFpEF.	Inflammation directly contributes to HF pathogenesis.
Alexandre et al., 2025 [20]	Prospective cohort/echocardiography study	277 RA patients; 24 months	In total, 24% demonstrated subclinical LV dysfunction despite preserved EF.	Subclinical myocardial injury and HFpEF phenotype detectable early.
Chandrupatla & Singh, 2025 [26]	National database study on HF prognosis	3,718,425 total HF admissions analyzed (RA vs. non-RA comparison); 32.7% were readmitted within 90 days total	Higher 90-day readmission (aOR 1.16) and mortality (aOR 1.12) in RA HF patients.	HF in RA is prognostically worse.

Abbreviations: RA, rheumatoid arthritis; HF, heart failure; HFpEF, heart failure with preserved ejection fraction; HFrEF, heart failure with reduced ejection fraction; aHR, adjusted hazard ratio; aOR, adjusted odds ratio; CRP, C-reactive protein; LV left ventricle; EF, ejection fraction.

**Table 2:** Therapeutic effects of RA treatments on heart failure risk (different levels of evidence for specific drug classes).

Therapy Class	HF Effect	Mechanistic Rationale	Key Supporting Studies
Methotrexate	Favorable/associated with lower HF risk	Inflammation suppression; endothelial/matrix modulation	Sun 2021 [41]; Johnson 2021 [16]; Cervantes 2024 (abstract) [42]
Hydroxychloroquine	Neutral/associated with higher HF-related events in selected high-risk populations	Mixed HF effects; QT concerns in high-risk HF	Sorour 2021 [43]; D’Andrea 2022 [44]; Iyer 2024 [45]
Glucocorticoids	Adverse (dose-dependent)	Metabolic toxicity, fluid retention, hypertension	Pujades-Rodriguez 2020 [40]; Ocon 2021 [17]
TNF inhibitors	Mixed/possibly protective/possibly harmful in advanced HF	TNF suppression improves vascular/immune milieu	Chen 2021 [48]; Galajda 2025 [49]; Solomon 2013 [51]
IL-6 inhibitors	Neutral	Inflammation reduction without HF-specific signal	Kim 2017 [53]; Castagné 2019 [54]
Abatacept	Possibly protective	T-cell modulation favoring CV risk reduction	Jin 2018 [66]
Rituximab	Neutral (caution in HF)	B-cell depletion; limited HF data	Van Vollenhoven 2013 [57]; Winthrop 2018 [58]
Anakinra	Neutral	IL-1 blockade; minimal HF data	Mertens 2009 [63]
JAK inhibitors	Neutral/caution in CV risk and HF	Immune modulation; signals vary by agent	Ytterberg 2022 [69]; Fleischmann 2023 [68]

Abbreviations: HF, heart failure; TNF, tumor necrosis factor; IL, interleukin; CV, cardiovascular; JAK, Janus kinase.

## Data Availability

No new data were created or analyzed in this study.
